# STK4 regulates TLR pathways and protects against chronic inflammation–related hepatocellular carcinoma

**DOI:** 10.1172/JCI193453

**Published:** 2025-04-15

**Authors:** Weiyun Li, Jun Xiao, Xin Zhou, Ming Xu, Chaobo Hu, Xiaoyan Xu, Yao Lu, Chang Liu, Shengjie Xue, Lei Nie, Haibin Zhang, Zhiqi Li, Yanbo Zhang, Fu Ji, Lijian Hui, Wufan Tao, Bin Wei, Hongyan Wang

Original citation: *J Clin Invest*. 2015;125(11):4239-4254. https://doi.org/10.1172/JCI81203

Citation for this corrigendum: *J Clin Invest*. 2025;135(8):e193453. https://doi.org/10.1172/JCI193453

The authors recently became aware that in Figure 8D of the original article, the two representative images for the *Stk4^ΔM/ΔM^* group were different crops of the same image. The correct panel, showing independent biological replicates provided from the original source data, is shown below:

## Figures and Tables

**Figure 8:**
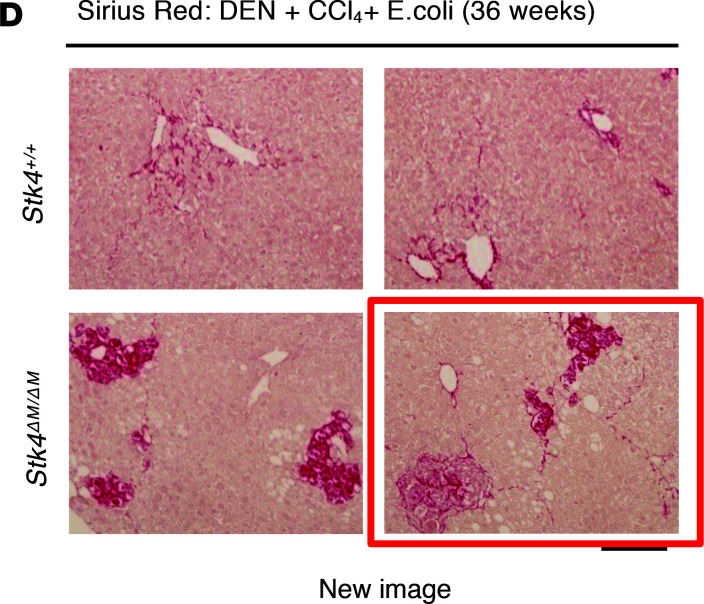
STK4 protects mice from DEN and bacterial infection-induced HCC in vivo.

## Version 1 04/15/2025

Electronic publication

